# Numerical Simulation, Machining and Testing of a Phase Change Heat Sink for High Power LEDs

**DOI:** 10.3390/ma12132193

**Published:** 2019-07-08

**Authors:** Jianhua Xiang, Haoxing Zheng, Yipin Wang, Chunliang Zhang, Chao Zhou, Conggui Chen

**Affiliations:** School of Mechanical and Electrical Engineering, Guangzhou University, Guangzhou 510006, China

**Keywords:** phase change heat sink, fabrication, numerical simulation, heat transfer testing, high power LED

## Abstract

Thermal management is crucial to guarantee the normal operation of light-emitting diodes (LEDs) Phase change heat sink is superior to traditional metal solid heat sink due to very small thermal resistance. In this study, a new type of phase change heat sink for high power LEDs is first designed. Then, the fabrication process of boiling structures at the evaporation surface of the phase change heat sink is discussed and analyzed. To make a comparison and deep discussion, the machining process is simulated through the FEM (finite element analysis) software, DEFORM-3D. Last but not least, heat transfer performance of the fabricated phase change heat sink is tested. Results have shown that the designed new type of phase change heat sink has superior heat transfer performance and is suitable for heat dissipation of high-power LEDs.

## 1. Introduction

Different from traditional incandescent lamps, fluorescent lamps and halogen lamps, light-emitting diode (LED) lamps are made of semiconductor materials. Light is generated by hole-electron pairs in a PN Junction. At present, the photoelectric conversion efficiency is between 30% and 40%, and almost all the rest of the electrical energy is converted into heat, resulting in the increase of the temperature of the LED. Generally, the LED chip size is smaller than 2.5 mm × 2.5 mm, namely the chip has very high heat flux. The heat must be dissipated to ensure the stability of the LED chip. In known LED failure cause, 55% were caused by high temperature [1]. As the temperature of the LED light source rises every 10 °C, the reliability reduces by half [2]. Therefore, effective heat management of the LED light source is really necessary to guarantee the normal operation of the LEDs [3,4].

The main point of thermal management is to keep LED working temperature within a reasonable range by heat transfer and heat dissipation. With regard to heat transfer, thermal conductive medium is applied to transfer the heat generated by the heat source to a specific location. The conductive medium should have high thermal conductivity, short heat flow channel and high heat transfer efficiency. When it comes to heat dissipation, convection and radiation are often applied to dissipate heat into the air. In terms of LED, heat is first transferred to finned heat sink through thermal conductive medium as heat pipe, and then out of the heat sink by heat convection [5].

Much research has been directed to phase change material heat sinks due to their excellent heat transfer performance [6,7,8,9]. Representatively, heat pipe is a kind of heat transfer unit with very high thermal conductive performance. It works by the effect of evaporation and condensation of the liquid in the vacuum tube (phase change heat transfer). The heat conduction capacity of heat pipe is higher than any known metal [10,11,12,13]. Heat pipe is often composed of main-body, wick and end-cover. The inside of the heat pipe is pumped to a negative pressure state, and then filled with an appropriate liquid, having a low boiling point and likely to undergo volatilization. The wick is in the pipe wall, and made up of capillary porous materials. One side of the heat pipe is the evaporation section (hereinafter referred to as the hot side), and the other side is the condensation section (hereinafter referred to as the cold side). When the hot side of the heat pipe is heated, the working liquid evaporates rapidly, and the steam flows to the cold side under the effect of pressure difference. When the steam gets to the cold side, it condenses into liquid and releases heat. Then, the liquid flows back to the hot side along the porous material by the effect of capillary force. The process repeats in this way, and the continuous transfer of heat from the hot side to the cold side can be realized. The heat can be transferred out of the heat source very quickly.

Capillary structure is the core of the heat pipe products [14]. It has three purposes: one is to provide the reflux channel to the liquid in the cold side, the second is to provide the channel to heat conduction between the wall and the liquid/vapor, and the third is to produce the porosity to the capillary pressure that is required. The capillary structure is often classified as four kinds: wire mesh, groove, powder sintering, and fiber. When it comes to the LED heat sink, groove and powder sintering are two of the most commonly used structures. For the sintering-type heat pipe, the capillary structure is made of copper powder through high temperature sintering. For groove-type heat pipe, an integral forming process is applied at manufacturing. The cost of integral forming process is about two-thirds of that of powder sintering. It should be pointed out that, although the heat can be transferred out of the heat source by heat pipe, the heat cannot be effectively dissipated to the surrounding environment due to the small cooling area. Therefore, fin structures are often applied to effectively transfer heat to the air. With the combination of heat pipe and fin structures, heat transfer and heat dissipation can be completed most effectively.

Natural convection is commonly used in the cooling way of LED lamps and lanterns [15,16,17,18,19]. With the increase of thermal conductivity and cooling area of the radiator, the temperature distribution of LED lamps and lanterns can be improved. Generally, the radiator of LED lamps and lanterns is made of aluminum alloy through die casting, extrusion and cold forging process. Copper alloy is sometimes used for the radiator. However, since the density of the copper alloy is large, the texture is soft and the production process is complicated, the weight and the cost of the copper-alloy radiator is high. Recently, magnesium alloy material has attracted much attention. The density and the heat capacity of magnesium alloy is smaller than those of aluminum and copper alloy, resulting in a higher heat transfer velocity and a better heat dissipation performance. Moreover, graphene has excellent thermal conductivity and has been researched and applied in the new type of heat radiator [20,21,22].

In this study, a new type of phase change heat sink for high power LEDs is designed and fabricated. As key structures related to heat transfer performance, the machining process of boiling structures at the evaporation surface of the phase change heat sink is numerically simulated and deeply discussed. Last but not least, heat transfer performance of the fabricated phase change heat sink is tested. Results have shown that the designed new type of phase change heat sink has superior heat transfer performance and is suitable for heat dissipation of high-power LEDs.

## 2. The Structure Design of Phase Change Heat Sink

**Figure 1:**
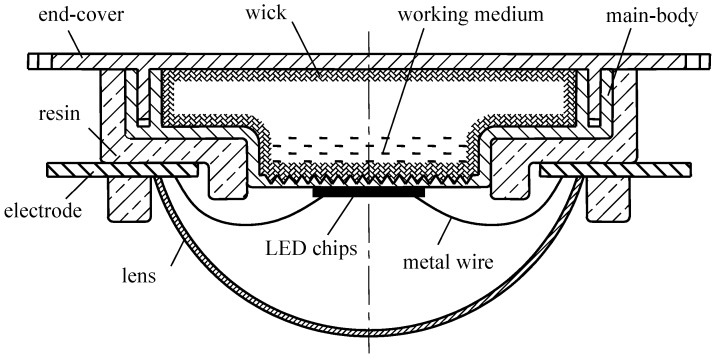
The schematic to the packaging of high-power LED with phase-change heat sink.

As it is shown in Figure 1, the phase change heat sink is inserted into LED system together with LED chips, resin, electrode, metal wire and lens etc. The heat generated by high power LED can be transferred out of the source in time through the phase change heat sink. The phase change heat sink is made up of main-body, end-cover, wick and the working medium. An airtight cavity is formed after the welding and encapsulation of main-body and end-cover. Then, the cavity is filled with liquid working substance and pumped into vacuum state. Porous materials inside the wick subsequently provides capillary force for the backflow of the liquid working medium.

The manufacture of phase change heat sink involves machining of main-body and end-cover, the formation of three-dimensional boiling enhancement structures, sintering of wick and perfusion of liquid working medium etc. As the foundation of high-power LED phase change heat sink structure, main-body and end-cover not only serve as the heat transfer interface, but also offer airtight space. When it comes to material selection, coefficient of thermal expansion of main-body and end-cover should be as close as possible to the LED chip so as to improve the reliability of high-power LED. Moreover, to achieve good welding performance, the material for main-body and end-cover should be consistent. In addition, the material for main-body and end-cover should satisfy the compatibility with liquid working medium. From the above, copper is selected as the material of heat sink main-body and end-cover.

**Figure 2:**
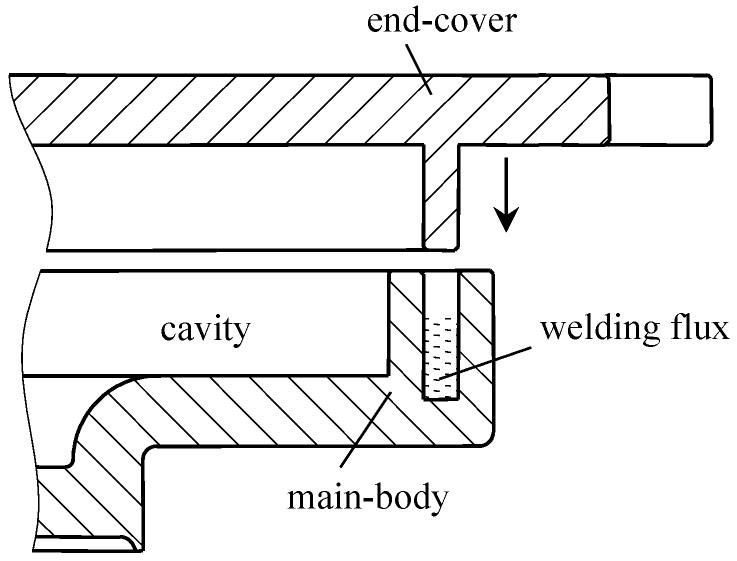
The schematic to the sealing of main-body and end-cover.

The schematic to the sealing of main-body and end-cover is shown in Figure 2. A sealing groove is first machined into the main-body. The welding flux is then filled into the groove. Subsequently, the end-cover and the main-body are assembled as shown in the schematic. After that, the solder is heated to the melting point to realize the sealing of the main-body and end-cover. Accordingly, an airtight cavity is formed.

To ensure the reflux of working medium, the wick should be formed around the cavity. Generally, very small pore radius is required to provide maximum capillary pressure, large permeability is necessary to reduce pressure loss in the reflux, and small thermal resistance is needed to better the radial heat transfer performance. In consideration of such factors as compatibility, sintered copper powder is applied to form the wick structure. The wick is formed by the sintering of a deal of metal powder in the wall. Since the copper has a high thermal conductivity and good sintering performance, copper powder is used for the formation of the wick structure in this study.

Phase change between liquid and steam is applied to the transfer of heat. Accordingly, physical properties of the working medium have an effect onto the working characteristic of phase change heat sink. To the selection of working medium, the medium should adapt to the highest temperature in the high-power LED workspace and have an appropriate saturation vapor pressure. Plus, the working medium should be compatible with the material in shell wick, and has good thermal stability and good comprehensive thermal physical properties. As water and ethanol are compatible with metallic copper, and the highest junction temperature of high-power LED is below 120 °C, pure water and ethanol are selected as the working medium. 

## 3. The Formation of Boiling Structures in Phase Change Heat Sink

To improve the boiling efficiency of the hot side thereby to reduce the thermal resistance of phase change heat sink, the formation process of boiling structures at the hot side is determinant to the heat transfer performance of the heat sink. As early as 1931, it was found that nucleate boiling can be improved through the rough surface. In recent years, many researchers have studied active or passive methods to improve the efficiency of boiling. In terms of active methods, electromagnetic fields and vibration methods are used to improve the efficiency of boiling. Nevertheless, due to the cost and reliability, active methods are less used than passive methods. In terms of passive methods, surface appearance is optimized to improve the boiling efficiency. Commonly used surface appearance includes various grooves, ribs and a porous surface. From the point of processing methods, there are mechanical machining, laser processing, sand blasting, flame spraying and sintering processes etc. 

Ploughing-extrusion, as a kind of green manufacturing method, is applied to form boiling enhancement structures at the hot side. Spiral grooves are first machined in the circular plane of the evaporation end, to serve as the flow channel to the working medium in the circumferential direction. Stamping method is then applied to machine radial grooves on the basis of spiral grooves, eventually to form three-dimensional microgrooves structures. In this kind of microgrooves, the radial and circumferential are connected with each other, not only providing capillary force to the liquid working medium, but also to enhance the boiling effect and improve the performance of evaporation.

### 3.1. Ploughing-Extrusion to Form Circumferential Spiral Grooves

**Figure 3:**
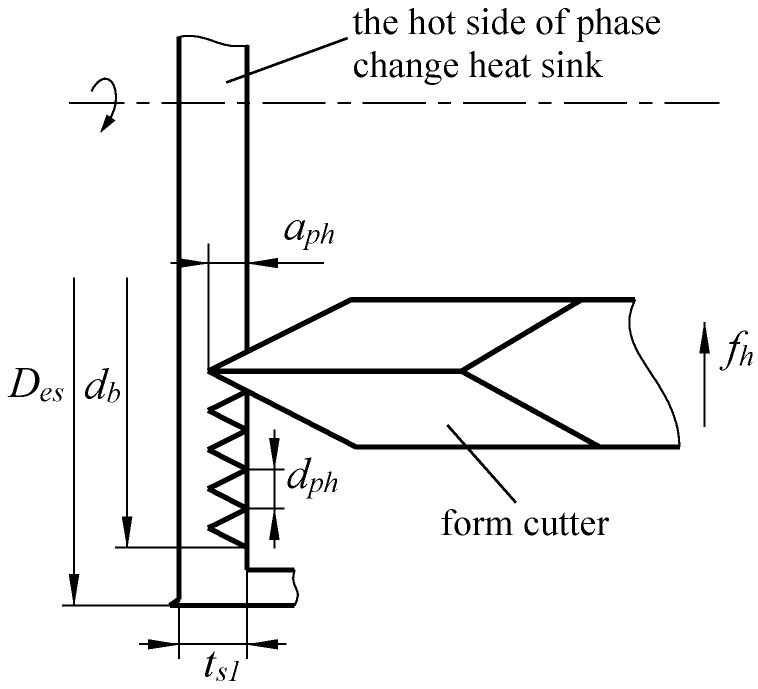
The schematic to form spiral grooves by P-E ploughing-extrusion processing.

Circumferential spiral grooves are formed at the hot side of phase change heat sink using common lathe upon special clamping. As it is shown in Figure 3, the workpiece is fixed by lathe chuck, and the cutter is installed in the direction parallel with the axis of the workpiece. The feeding is along the radial direction. With the rotary motion of the spindle, the feeding of the cutter is set to a constant value, then the spiral microgrooves can be formed at the inner surface of the evaporation side. In Figure 3, the D_es_ is the outer diameter of the evaporation side, *d*_b_ is the diameter of boiling structures, *a*_ph_ is the depth of ploughing-extrusion (P-E), *d*_ph_ is the slot pitch of ploughing-extrusion, and *f*_h_ is the feeding of ploughing-extrusion.

### 3.2. Stamping to form Radial Boiling Structures

**Figure 4:**
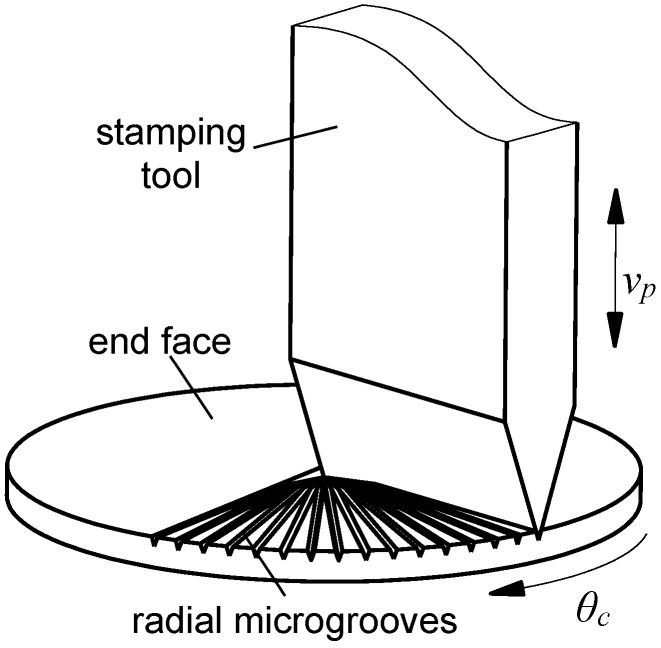
The schematic to form radial boiling structures.

In this study, stamping method is applied to machine radial microgrooves at the bottom of the hot side. As it is shown in Figure 4, the workpiece is fixed on the rotating disk, and the stamping tool is installed in the direction parallel with the axial direction of the workpiece. Given a certain stamping depth, the cutting tool moves downward to stamping the workpiece, and a radial groove is formed. Then the cutting tool moves upward, and at the same time, the rotating disk turns a certain angle. Subsequently, the cutting tool moves down again to form another microgroove. The angle between two neighbor microgrooves is equal to the rotation angle of the rotating disk. The cutting tool then reciprocates up and down, not until the entire radial boiling microgrooves are formed whose origin is located at the centre of the workpiece. This kind of structure is advantageous to liquid working medium flow from the radius to the center.

### 3.3. The Analysis of Ploughing-Extrusion Process

**Figure 5:**
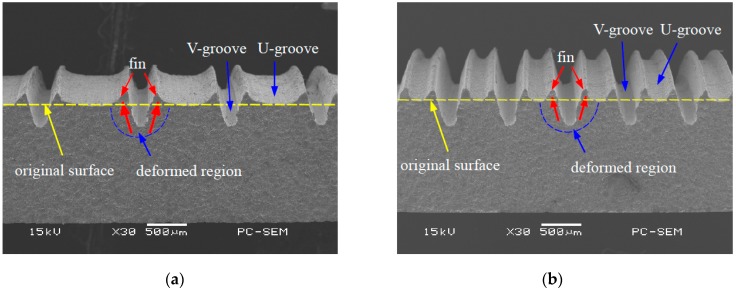
Mixed structures of V-grooves and U-grooves by P-E processing. (**a**) *d*_ph_ = 0.80 mm, *a*_ph_ = 0.20 mm; (**b**) *d*_ph_ = 0.40 mm, *a*_ph_ = 0.20 mm.

**Figure 6:**
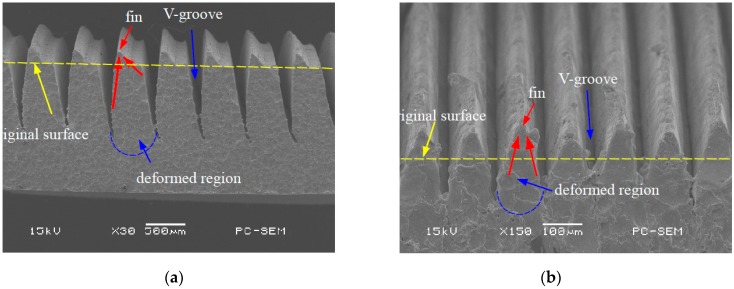
V-grooves by P-E processing. (**a**) *d*_ph_ = 0.20 mm, *a*_ph_ = 0.20 mm; (**b**) *d*_ph_ = 0.16 mm, *a*_ph_ = 0.20 mm.

Ploughing-extrusion is performed with different ploughing-extrusion depths and feedings. The SEM (scanning electron microscope) images of the morphology of microgrooves boiling structures are shown in Figure 5 and Figure 6. From the machining process, the pitch *d*_ph_ is determined by the feeding. Figure 5a shows the morphology of microgrooves when *d*_ph_ = 0.80 mm and *a*_ph_ = 0.20 mm. It can be seen from the figure that the bulge at V-groove edge is small, with a height of about 45 μm. To compare Figure 5b with 5a, it is found that the bulge at V-groove edge increases with the decrease of the *d*_ph_. Figure 5b displays the morphology of microgrooves when *d*_ph_ = 0.40 mm and *a*_ph_ = 0.20 mm, in which the height of the bulge is about 60 μm. Moreover, *d*_ph_ and *a*_ph_ have a significant effect onto the formation of fin structures. When *d*_ph_ is larger or *a*_ph_ is smaller, U-grooves are formed by the bulges between two neighboring V-grooves. Accordingly, mixed structures of V-grooves and U-grooves are formed.

Figure 6a exhibits the morphology of microgrooves when *d*_ph_ = 0.2 mm and *a*_ph_ = 0.2 mm. It can be seen that the bulges at V-groove edge are cut off, and so triangular fins with sharp top are formed between two neighboring V-grooves. The height of the fin is about 0.40 mm. When *d*_ph_ decreases, single V-groove structure with sharp top is formed, as the morphology of microgrooves that is shown in Figure 6b when *d*_ph_ = 0.16 mm and *a*_ph_ = 0.2 mm. With the comparison of Figure 6a,b, the height of the fin will be decreased when *d*_ph_ further reduces.

## 4. Numerical Simulation

### 4.1. Material Properties

The material of the workpiece is copper with a diameter of 10 mm and a thickness of 1 mm. In the process of ploughing-extrusion and stamping, plastic strain was generated. Physical parameters of the workpiece are shown in Table 1.

### 4.2. Finite Element Model

**Figure 7:**
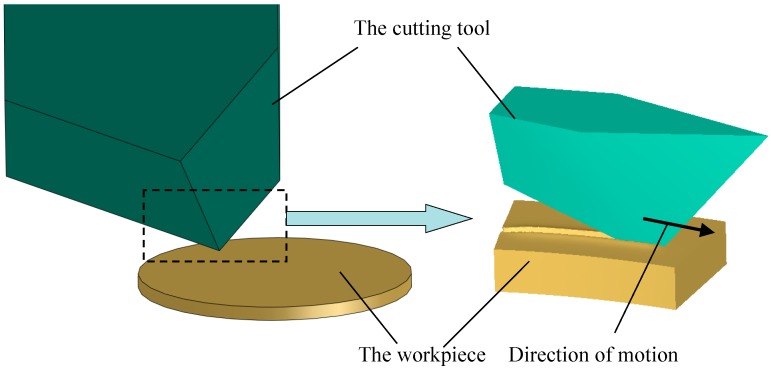
Geometry model of the workpiece and the cutting tool in simulation.

To simulate the ploughing-extrusion process through the FEM software DEFORM-3D v11.0, the workpiece is deemed as a fixed part, whereas, the cutting tool is deemed as a rotation part. The axis of rotation is at the centre of the workpiece rotating with a certain speed so as to realize the relative movement between the workpiece and the cutting tool. As the feed and spiral angles are different, the following simplification is made in finite element calculation, i.e., every two adjacent grooves are machined during two different forming process. Due to the axisymmetric characteristic of the workpiece, only 1/8 of the workpiece was taken out for simulation. The selected workpiece geometry model is exhibited in Figure 7.

**Figure 8:**
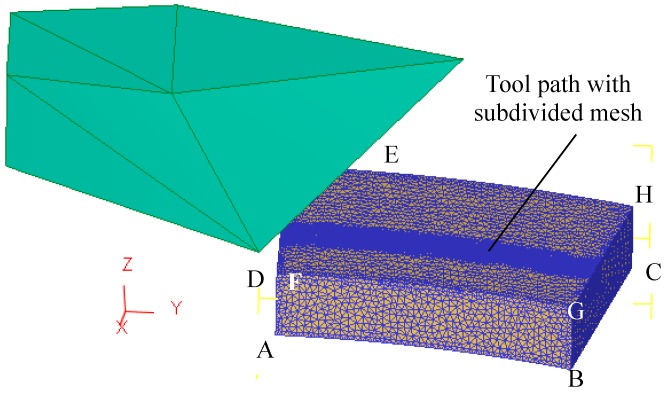
Finite element mesh model of the workpiece.

The cutting tool is deemed as a rigid body, while the workpiece is deemed as a rigid-plastic body. First of all, the workpiece was divided into 100,000 tetrahedron elements. Subsequently, the contact path between the cutting tool and the workpiece was further subdivided, as is shown in Figure 8. Due to the large plastic deformation of the workpiece during processing, adaptive re-meshing was adopted to avoid the excessive distortion of mesh. During the simulation, the ploughing-extrusion depth was set from 0.1 to 0.5 mm.

As the deformation on the back of the evaporation end is very small during processing, six degrees of freedom of the bottom of the workpiece is set to zero during simulation, namely the xyz moving directions and xyz rotation directions. Plus, the displacement in z direction at the surface of ABGF and DCHE is set to zero, in order to simulate the forming of the fin structures and the micro grooves. During simulation, the friction model is simplified as follows,

(1) $$F_{f}=\mu \cdot [\tau ]\cdot A_{f}$$

where *F_f_* is friction, *μ* is a constant value of 0.12, *A_f_* is the contact area between the cutting tool and the ploughing-extrusion surface.

### 4.3. Results and Discussions

**Figure 9:**
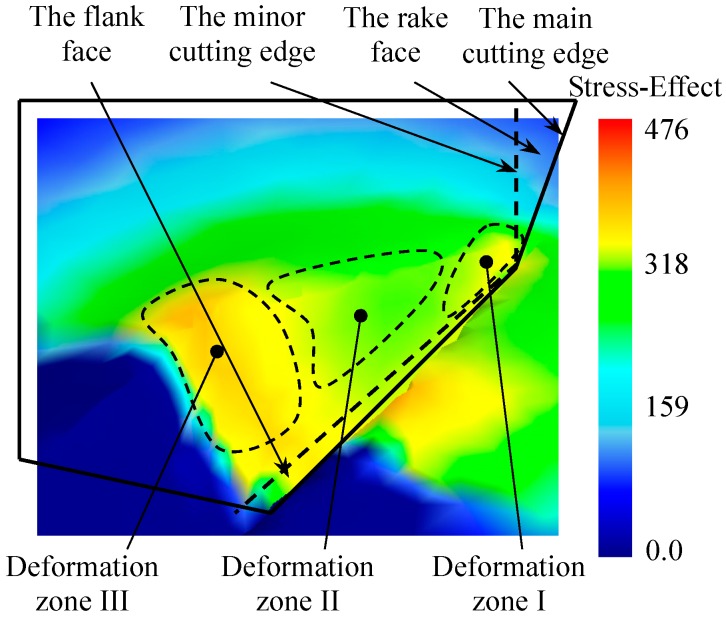
Deformation zones in the process of fin forming.

In order to analyze the process of fin forming, the metal layer is divided into three deformation zones according to different deformation patterns, as is shown in Figure 9. Deformation zone I is the plastic zone produced by the main cutting edge and the rake face of the cutting tool in the metal layer. Deformation zone II is produced by the minor cutting edge and the rake face of the cutting tool. Deformation zone III is the deformation area inside the forming micro grooves, where work hardening occurs under the effect of the extrusion and friction of the forming plane.

**Figure 10:**
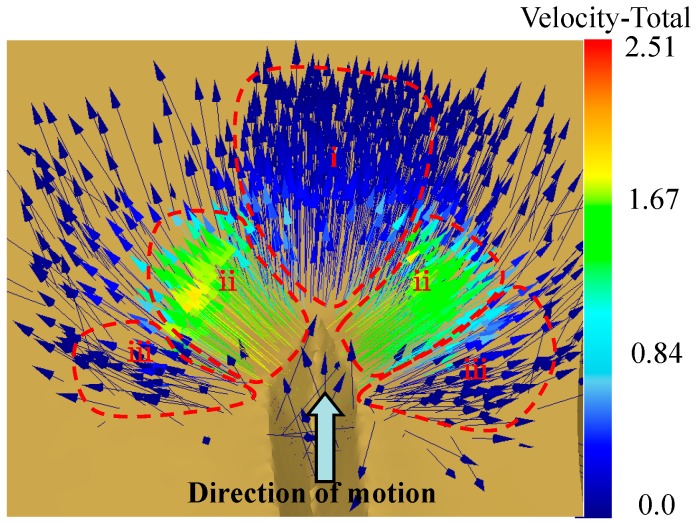
The flow vector of the metal during the process.

During the process of ploughing-extrusion, the blade first contacts with and then comes into the surface of the workpiece gradually. Under the extrusion of the main cutting edge and the rake face of the cutting tool, plastic deformation is generated in deformation zone I. According to the law of least resistance, the materials move to the direction with least resistance. Therefore, the metal moves to the unconstrained upper surface of the workpiece at the same time while moving forward. The moving speed is inversely proportional to the distance to the rake face of the cutting tool. After the surface layer of the metal is cut open, in deformation zone II, the split metal starts to flow to the main and auxiliary extrusion surface separately to form micro grooves under the effect of extrusion. A mass of metal is squeezed up that produces uplift near the cutting edge. The metal at the bottom and in the middle of the grooves flows separately and gets to the edge of the groove. In deformation zone III, the metal continues to flow to the two sides of the cutting tool under the extrusion of the forming plane, resulting in the formation of the fin structure. The flow direction of the metal is shown in Figure 10.

**Figure 11:**
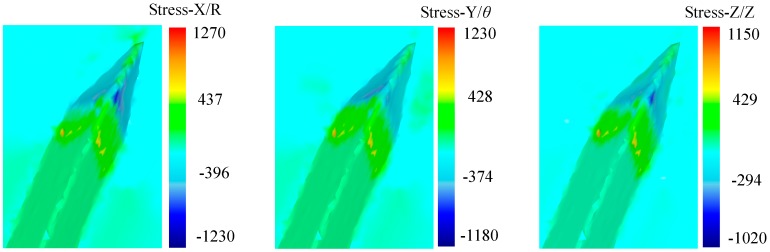
Tri-axial stress distribution in the process.

As is shown in Figure 11, tri-axial stress is compressive around the main cutting edge and the rake face of the cutting tool due to the extrusion effect. The value of the stress increases gradually along the groove depth direction. On the other hand, in the area around the forming plane of the cutting tool, tri-axial stress is tensile due to the effect of friction and adhesion effect between the metal and the forming plane. While in the noncontact zone, tri-axial stress is compressive by the extrusion of adjacent metal. 

Effect of different feedings onto the morphology of microgrooves was then investigated. To facilitate research, the ploughing-extrusion depth is set to a constant value, i.e., 0.1 mm in this study. In the simulation, a strip of microgroove was first fabricated with a ploughing-extrusion depth of 0.1 mm. Subsequently, another strip of microgroove was simulated with different feedings as 0.05 mm, 0.1 mm, 0.2 mm, 0.5 mm, respectively.

**Figure 12:**
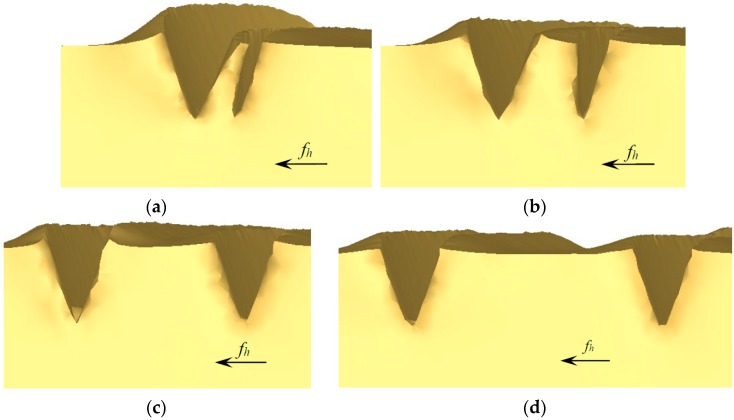
The morphology of microgrooves under different feedings in simulation. (**a**) *A*_ph_ = 0.1 mm, *f*_h_ = 0.05 mm; (**b**) *a*_ph_ = 0.1 mm, *f*_h_ = 0.1 mm; (**c**) *a*_ph_ = 0.1 mm, *f*_h_ = 0.2 mm; (**d**) *a*_ph_ = 0.1 mm, *f*_h_ = 0.5 mm.

The morphology of microgrooves with different feedings are shown in Figure 12. When the feeding is small, the first strip of microgroove is significantly influenced by the second strip of microgroove, i.e., the wall close to the second strip tilt out by extrusion. So, the width of the first strip of microgroove is reduced. Moreover, as a part of fin was cut off, the height of the fin decreases, making the top very sharp. In case of the second strip of microgroove, the height and the volume both increase, because the former cut-off part of the fin piles up onto the second fin, as is shown in Figure 12a. When the feeding is further reduced, the flash tends to occur due to the excessive accumulation of metal cut off from the first strip of microgroove. Thus, the feedings should not be too small.

With the increase of the feeding, the width of the first strip of groove increases as well, but still smaller than the original. As the inclined wall of the first strip of microgroove that is close to the second strip is not fully cut off, a U-shape microgroove is formed, resulting in the mixture of U-shape and V-shape grooves, as were displayed in Figure 12b,c, respectively. When the feeding further increases, there is little influence of the second strip of groove onto the first strip, i.e., no interference with each other, as is exhibited in Figure 12d with a feeding of 0.5 mm. In this regard, the forming mechanism is consistent with single groove formation mechanism.

**Figure 13:**
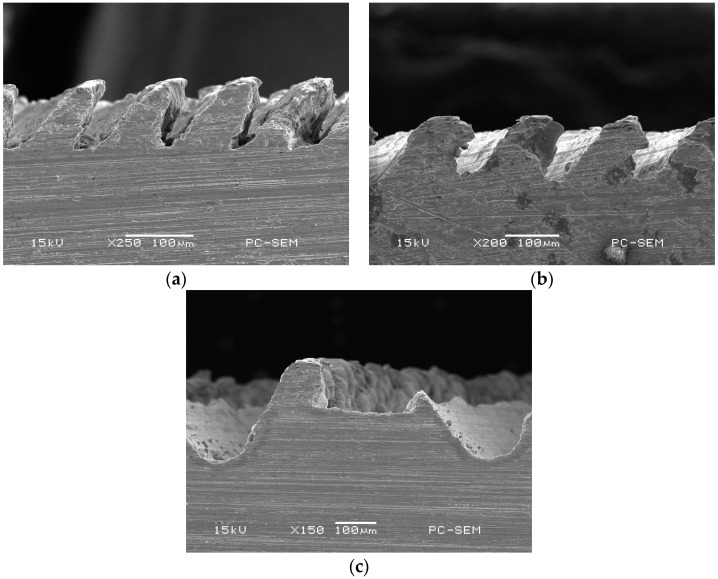
SEM section images of microgrooves under different *d*_ph_ in experiment. (**a**) *a*_ph_ = 0.1 mm, *d*_ph_ = 0.21 mm; (**b**) *a*_ph_ = 0.1 mm, *d*_ph_ = 0.31 mm; (**c**) *a*_ph_ = 0.1 mm, *d*_ph_ = 1.24 mm.

To verify the simulation, the microgroove structure was machined in experiment with its morphology shown in Figure 13. In the machining process, the value of *d*_ph_ is proportional to the feeding *f*_h_. So, the morphology of microgrooves with different *d*_ph_ are machined and compared with that of simulation. It can be seen that, Figure 13a corresponds to Figure 12a, where the wall tilts out by extrusion. Figure 13b corresponds to Figure 12b,c, where rectangular fin structure is formed. Figure 13c corresponds to Figure 12d, where the width of the U-shape groove increases as the feeding. In brief, the simulation results are consistent with the experimental results. 

## 5. Heat Transfer Performance Testing of the Phase Change Heat Sink

**Figure 14:**
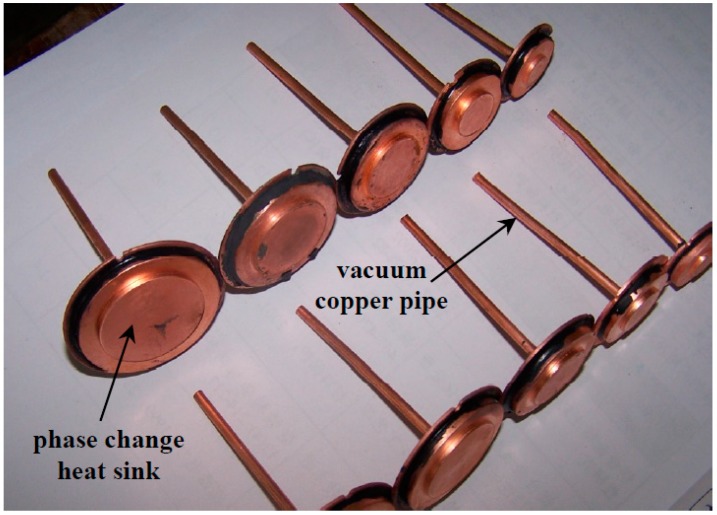
The phase change heat sink specimens.

**Figure 15:**
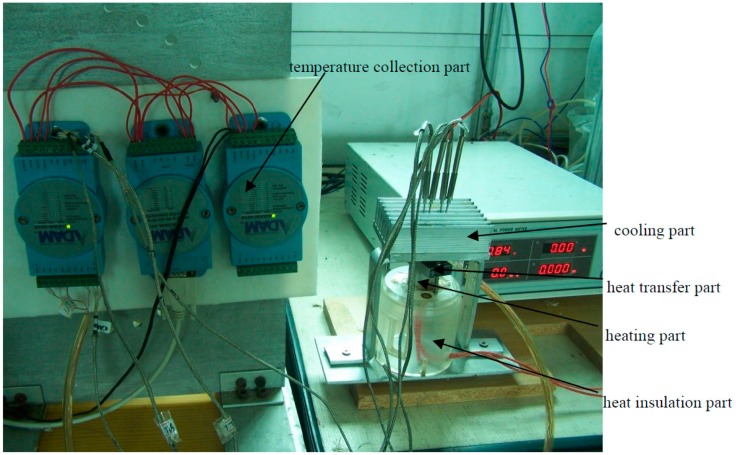
The heat transfer performance testing system.

The photograph of fabricated specimens of phase change heat sink are shown in Figure 14, which is instrumented with the heat transfer performance testing system shown in Figure 15. It is seen from Figure 15 that the testing system consists of the heating part, the heat insulation part, the heat transfer part, the cooling part and the temperature collection part. To collect testing results as accurately as possible, insulating materials are used to wrap the phase change heat sink and the heating source to avoid heat loss. At the same time, in order to improve the reliability of the LED, natural convection cooling is adopted rather than liquid or forced air cooling. To simulate natural convection, finned aluminum blocks are installed onto the end-cover of the phase change heat sink. The contact surface is polished and coated with a small amount of thermal conductive silica gel, to reduce thermal contact resistance.

**Figure 16:**
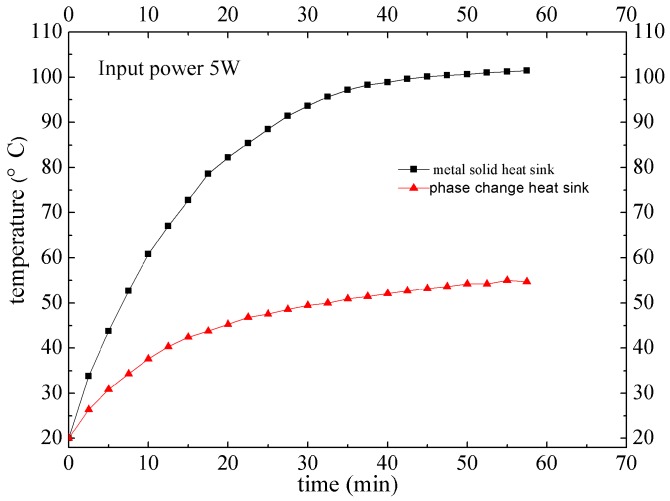
Heat transfer performance of phase change heat sink vs. metal solid heat sink.

With different input power as 3 W, 5 W and 10 W, heat transfer performance of the phase change heat sink and metal solid heat sink was tested, respectively. It can be seen from Figure 16 that, when the input power is 5 W, it takes about 50 min to get to the balance. The highest temperature of the phase change heat sink is 54.7 °C, while the highest temperature of traditional metal solid heat sink is 101.7 °C. In consideration of thermal contact resistance, encapsulation thermal resistance and thermal resistance inside LED chip, the LED junction temperature most likely overpasses the highest temperature of 120 °C. Consequently, metal solid heat sink is commonly used when the input thermal power is below 3 W.

**Figure 17:**
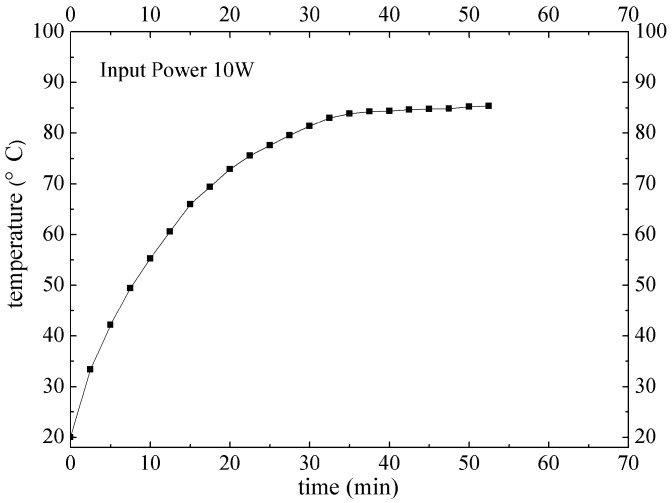
Heat transfer performance of phase change heat sink under the input power of 10 W.

Heat transfer performance of phase change heat sink under the input power of 10 W is shown in Figure 17. It takes about 40 min to get to the balance. The highest temperature of the phase change heat sink is 85.4 °C, far below the allowing highest LED junction temperature of 120 °C. Accordingly, the phase change heat sink developed in this study has very good heat transfer performance, and is suitable for LED packaging under the input power of 10 W. To make a comparison, thermal resistance of the phase change heat sink is 0.19 °C/W, i.e., 1/2 of that of the metal solid heat sink (0.38 °C/W).

## 6. Conclusions

This paper proposed a new type of phase change heat sink for high power LEDs. The fabrication process of the phase change heat sink is numerically simulated and compared with that of the experiment. Heat transfer performance of the fabricated phase change heat sink is tested. Results have shown that heat transfer performance of the heat sink is much better than that of traditional metal solid. In addition, the developed new type of phase change heat sink is suitable for high power LEDs applications. 

## Figures and Tables

**Table 1:** Material properties of the workpiece.

Elasticity Modulus (GPa)	Poisson’s Ratio	Yield Stress (MPa)	Linear Dilatation Coefficient	Specific Heat Capacity J/(kg·K)	Heat Conductivity W/(m·K)	Mass Density (kg/m^3^)
118.5	0.3	227	1.7 × 10^−5^	3.42	400	8.96 × 10^3^
